# Vaccine-Induced Waning of *Haemophilus influenzae* Empyema and Meningitis, Angola

**DOI:** 10.3201/eid2011.140400

**Published:** 2014-11

**Authors:** Heikki Peltola, Tuula Pelkonen, Luis Bernardino, Lurdes Monteiro, Silvia da Conceição Silvestre, Elizabete Anjos, Manuel Leite Cruzeiro, Anne Pitkäranta, Irmeli Roine

**Affiliations:** Helsinki University Central Hospital and University of Helsinki, Helsinki, Finland (H. Peltola, T. Pelkonen, A. Pitkäranta);; David Bernardino Children’s Hospital, Luanda, Angola (T. Pelkonen, L. Bernardino, S. da Conceição Silvestre, E. Anjos, M.L. Cruzeiro);; National Health Institute of Lisbon, Lisbon, Portugal (L. Monteiro);; University Diego Portales, Santiago, Chile (I. Roine)

**Keywords:** *Haemophilus influenzae*, Africa, empyema, pneumonia, meningitis, bacteria, vaccine, Angola

## Abstract

In Angola during 2003–2012, we detected *Haemophilus influenzae* in 18% of 2,634 and 26% of 2,996 bacteriologically positive pleural or cerebrospinal fluid samples, respectively, from children. After vaccination launch in 2006, *H. influenzae* empyema declined by 83% and meningitis by 86%. Severe *H. influenzae* pneumonia and meningitis are preventable by vaccination.

In sub-Saharan Africa, most children with severe community-acquired pneumonia are discharged from care with the causative agent unidentified. In the few diagnosed cases, diagnosis is usually based on positive blood culture and, to a growing extent, positive urine antigen test result for *Streptococcus pneumoniae* (*1*). For meningitis diagnosis, cerebrospinal fluid (CSF) (and sometimes blood) samples are cultured routinely.

Acute pneumonia can result in fluid collection in the pleural space, a parapneumonic effusion visible on chest radiographs. Effusions tend to become purulent. Fluid removal (often >100 mL) by thoracentesis, along with appropriate antimicrobial drug therapy, relieves dyspnea, speeds healing, and sometimes saves lives. Identification of the causative agents gives a hint of the etiology of nontrivial forms of pneumonia. Following this rationale, we analyzed a large series of empyema and bacterial meningitis cases in children in Angola. We sought to discover to what extent *Haemophilus influenzae* type b (Hib) vaccination, started in June 2006, influenced occurrence of these potentially life-threatening diseases.

## The Study

Our prospectively collected data came from 23,134 children 2 months to 13 years of age during 2003–2012. All had attended Angola’s largest pediatric center, the 300-bed David Bernardino Children’s Hospital in Luanda, which has been the core of our treatment trials (*2*), all approved by the local ethics committee. From these children, 6,030 pleural fluid samples and 17,104 CSF samples were examined by microscopy, gram-stained, and cultured. Any child with symptoms and signs of pleural effusion and a large, not blade-like, opacity on chest radiographs underwent thoracentesis, with or without chest tube insertion, under local anesthesia. Some 200 children a year fall into this category (*3*), and the procedure has become routine among attending pediatricians or surgeons. 

In 2002, in collaboration with the National Health Institute of Lisbon, Portugal, the hospital established a basic bacteriology laboratory and provided staff training (*4*,*5*). Microscopy and gram-staining of each pleural or CSF sample were performed 24 hours a day (Ziehl-Neelsen staining on demand), whereas bacterial culture on blood and chocolate agar plates was conducted only during working hours (specimens maintained at room temperature). Culture techniques were standard. Lack of resources limited searches to aerobic pathogens.

Routine serotyping of *H. influenzae* and *S. pneumoniae* isolates was also beyond the reach of this institution. However, as routine hospital procedure, several hundred pleural fluid and CSF samples were sent to bacteriologists in Lisbon for further investigation (serotyping, PCR, some culture and susceptibility confirmation). Neither antigen-detection methods in Luanda nor PCR in Lisbon were used routinely, nor was HIV testing routinely performed in Luanda; however, in an earlier meningitis trial (*2*), we found that 8% of children in the study were HIV positive, in accordance with findings of a local epidemiologic survey (*6*). For patients with mixed bacterial infections, all agents were taken into account for analysis. Likely skin contaminants (5%) were excluded by a microbiologist (L.M.). No parasitologic or fungal investigations were conducted.

For most patients, the pleural fluid was purulent. After thoracentesis, pneumothorax or subcutaneous emphysema developed in a few patients but resolved spontaneously for all. The use of pretreatment antimicrobial drugs could not be determined, but because 271 (40%) of 679 children in our prospective meningitis study (*2*) were receiving antimicrobial drugs when they arrived at the hospital, pretreatment was likely.

In all, 2,634 (44%) of 6,030 pleural fluid samples yielded bacteria. As is characteristic of empyema patients (*3*), most were 7–23 months of age. *H. influenzae* was the third most common isolate (486 [18%]), exceeded only by *S. pneumoniae* (1,044 [40%]) and *Staphylococcus aureus* (690 [26%]). Thereafter came *Escherichia coli* (95 [4%]), streptococci other than pneumococcus (88 [3%]), *Proteus* spp. (87 [3%]), *Klebsiella* spp. (48 [2%]), and other gram-negative bacteria (96 [4%]). No *Mycobacterium tuberculosis* was detected.

**Figure 1:**
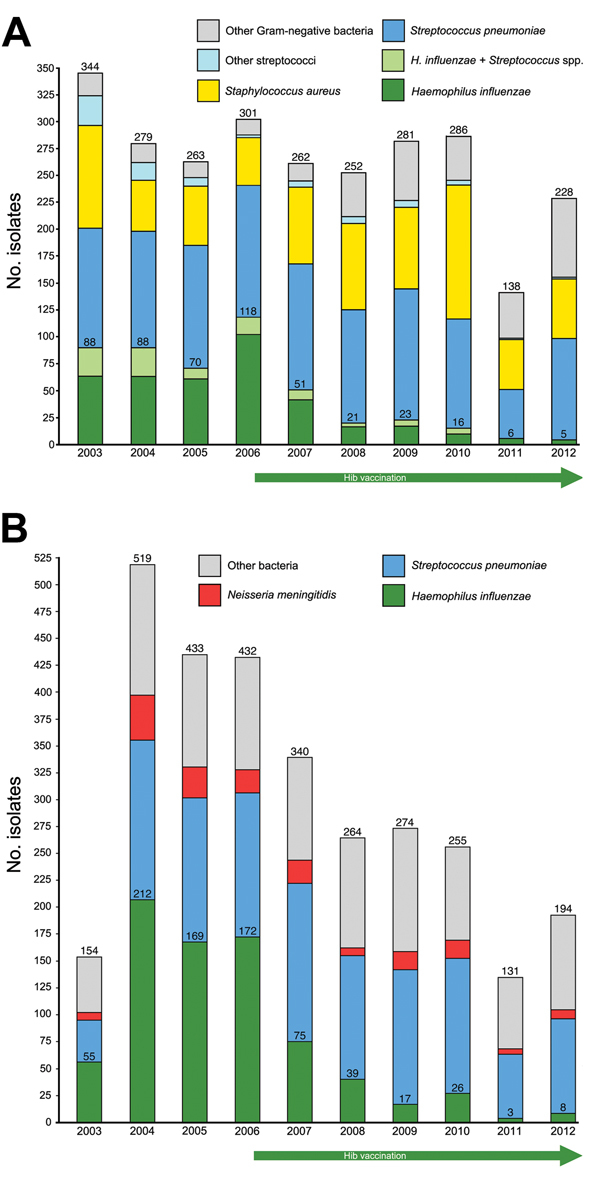
Distribution of 2,634 pleural fluid (empyema) (A) and 2,996 cerebrospinal fluid (CSF) isolates (B) from children who received treatment at David Bernardino Children’s Hospital, Luanda, Angola, during 2003–2012. Numbers above the light green bars in the upper panel comprise the total *Haemophilus influenzae* isolates found alone or with *Streptococcus* spp. (mostly *S. pneumoniae*). The numbers of mixed infections were as follows: 24, 24, 9, 15, 6, 2, 4, 6, 0, and 0, respectively. Hib, *H. influenzae* type b.

After vaccination launch in 2006, the number of *H. influenzae* isolations from patients with empyema clearly decreased (Figure 1). Although *H. influenzae*, sometimes concomitantly with pneumococci, was isolated 415 times during 2003–2007, it was isolated only 71 times during 2008–2012, an 83% decline. Serotyping was performed for 29 isolates, and all except 1 were Hib. Only 8 isolates of *S. pneumoniae* from patients with empyema were serotyped; distribution was 6A/B/C (n = 3), 1 (n = 2), 14 (n = 2), and 23F/A/B (n = 1).

After vaccination launch in 2006, the number of *H. influenzae* isolates from patients with meningitis also decreased (Figure 1). Of 17,104 CSF specimens, 2,996 yielded bacteria, of which 1,109 (37%) were *S. pneumoniae;* of 45 serotyped strains, the following serotypes were found: 6 (n = 14), 23 (n = 11), 1 (n = 6), 19F/A/B/C (n = 5), 14 (n = 3), 4 (n = 2), 18F/A/BC (n = 2), 3 (n = 1), and 5 (n = 1). *H. influenzae* (776 [26%]) was the second most common bacterial agent isolated. From the first 5-year period (683 cases) to the second period (93 cases), *H. influenzae* isolations decreased by 86%. All 55 serotyped *H. influenzae* strains were type b. No similar decreasing trend was found for *Streptococcus agalactiae,* 196 (7%); *Neisseria meningitidis,* 175 (6%); or gram-negative rods such as *E. coli,* 164 (5%), *Klebsiella* spp., 152 (5%), or *Salmonella* spp., 51 (2%).

**Figure 2:**
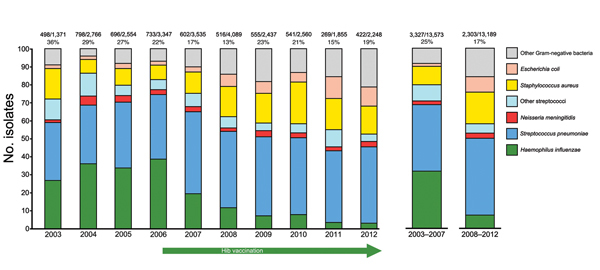
Proportional decrease in *Haemophilus influenzae* isolates starting in 2007 shows the effect of Hib vaccination launched in June 2006. Numbers above the bars indicate no. positive samples/no. cultured cultured samples (%) for all empyema and meningitis cases combined. Hib, *H. influenzae* type b.

Figure 2 depicts organisms isolated for cases of empyema and meningitis combined. All major changes occurred only for the empyema and meningitis caused by Hib, not for diseases caused by *S. pneumoniae* or other bacteria.

## Conclusions

Although empyema represents only a fraction of all cases of pneumonia among children in Angola, and only a few *H. influenzae* or *S. pneumoniae* isolates from pleural fluid and CSF were serotyped*,* the potential for decreasing pneumonia and bacterial meningitis by use of conjugate vaccines was demonstrated. The institution’s policy of performing thoracentesis for patients with empyema enabled us to examine the effects of Hib vaccination because the etiologic agent was determined for specimens obtained directly from the infection focus. A method much simpler and safer than chest tube insertion would have been transthoracic needle aspiration, for which risks are much exaggerated (*7*,*8*); regrettably, this reliable method is rarely used (*9*). When performed in the right place by the right clinicians on the right patients, it is a great tool for clinicians, patients, and epidemiologists.

That the bacterium most commonly isolated from pleural fluid was *S. pneumoniae* was not unexpected, nor was the large role of *S. aureus*, as documented in Africa, unexpected (*10*,*11*). The lesson learned was the high prevalence of Hib among patients with empyema. Because 79% (11/14) of cases of *H. influenzae* pneumonia in The Gambia were caused by Hib (*12*), and because a vaccination study in the same country provided strong evidence that Hib causes 21% of radiologically proven cases of pneumonia (*13*), the dramatic decline in Hib pneumonia and meningitis in Luanda fits the general picture well. Because by 2012, already 91% of vaccinees had received 3 doses, the vaccine effect was undeniable. A serologic study from China also showed Hib to be a major cause of childhood pneumonia (*14*). Obviously, not only empyema but also many other forms of pneumonia are preventable by Hib vaccination (*15*).

We are aware of study shortcomings. Because of economic constraints, only a fraction of the strains could be typed, and no *M. tuberculosis,* parasites, or viruses were sought. Still, our data showed that, besides meningitis, Hib is a major cause of severe pneumonia in Luanda. This vaccine-preventable disease is almost certainly not a problem specific to Angola; therefore, efforts should be made to implement Hib vaccination throughout Angola and elsewhere in sub-Saharan Africa. 
